# Supplementary material for: Zein-Based Films Containing Monolaurin/Eugenol or Essential Oils with Potential for Bioactive Packaging Application
Source: Int J Mol Sci. 2021 Dec 29;23(1):384. doi: 10.3390/ijms23010384 (PMC8745270; doi:10.3390/ijms23010384)
Supplement: Supplementary file 1 [file ijms-23-00384-s001.zip › ijms-1511233-supplementary.pdf]

Supplementary material to

Zein-based Films Containing Monolaurin/Eugenol or Essential Oils with Perspective for Bioactive Packaging

Jana Sedlarikova [1,*], Magda Janalikova [2,*], Pavel Pleva [2], Lucie Pavlatkova [2], Antonin Minarik [3, 4], and Petra Peer [2]

1 Department of Fat, Surfactant and Cosmetics Technology, Faculty of Technology, Tomas Bata University in Zlin, Vavreckova 275, 760 01 Zlin, Czech Republic

2 Department of Environmental Protection Engineering, Faculty of Technology, Tomas Bata University in Zlin, Vavreckova 275, 760 01 Zlin, Czech Republic

3. Department of Physics and Materials Engineering, Faculty of Technology, Tomas Bata University in Zlin, Vavreckova 275, 760 01 Zlin, Czech Republic

4. Centre of Polymer Systems, Tomas Bata University in Zlin, Trida Tomase Bati 5678, 76001 Zlin, Czech Republic

* Correspondence: sedlarikova@utb.cz; mjanalikova@utb.cz

In this supplementary material, the following figures are presented:

- Figure S1. SEM photographs of zein-based films with active compounds combinations: Z, zein; O, oregano; T, thyme; EU, eugenol; ML, monolaurin; 2: 2 wt%; 3: 3 wt%
- Figure S2. SEM photographs of zein-based films with active compounds combinations: Z, zein; O, oregano; T, thyme; EU, eugenol; ML, monolaurin; 2: 2 wt%; 5: 5 wt%
- Figure S3. Appearance of solvent cast zein-based films.

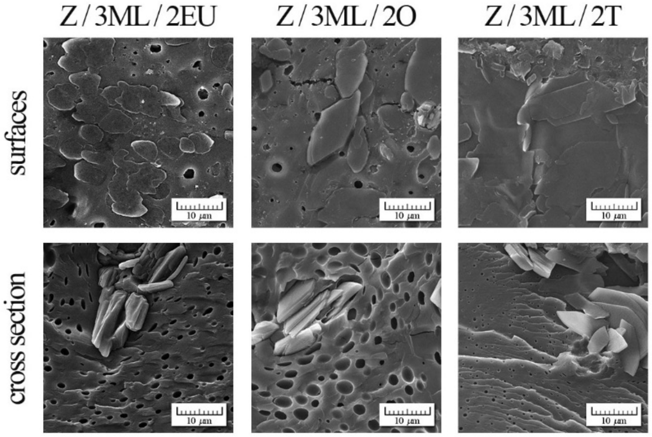

Figure S1. SEM photographs of zein-based films with active compounds combinations: Z, zein; O, oregano; T, thyme; EU, eugenol; ML, monolaurin; 2: 2 wt%; 3: 3 wt%

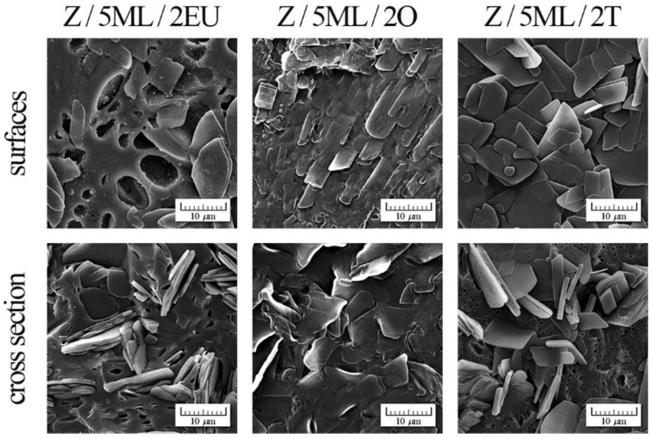

Figure S2. SEM photographs of zein-based films with active compounds combinations: Z, zein; O, oregano; T, thyme; EU, eugenol; ML, monolaurin; 2: 2 wt%; 5: 5 wt%

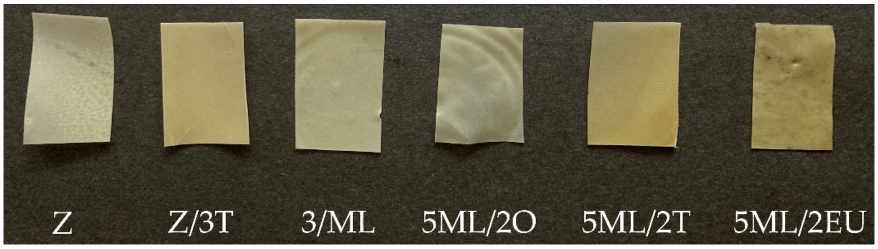

Figure S3. Appearance of solvent cast zein-based films.
